# Recurrent bilateral cutaneous squamous cell carcinoma arising within hypertrophic lichen planus with metastasis while on cemiplimab

**DOI:** 10.1016/j.jdcr.2022.08.046

**Published:** 2022-09-05

**Authors:** Melissa C. Leeolou, Nareh Marukian Burgren, Carolyn S. Lee, Arash Momeni, Harlan Pinto, Peter Johannet, Cara Liebert, Kristin M. Nord, Anne Lynn S. Chang

**Affiliations:** a Department of Dermatology, Stanford University School of Medicine, Redwood City, California; b Department of Dermatology, Veterans Administration Palo Alto Healthcare System, Palo Alto, California; c Department of Surgery (Plastic and Reconstructive), Stanford University School of Medicine, Palo Alto, California; d Department of Oncology, Veterans Administration Palo Alto Healthcare System, Palo Alto, California; e Surgical Service, Plastic Surgery Section, Veterans Administration Palo Alto Health Care System, Palo Alto, California

**Keywords:** cutaneous immune-related adverse event, cutaneous toxicity, hypertrophic lichen planus, hyponatremia, lichen planus, lichenoid, lymph node, metastasis, PD-1, PD-1 inhibitor, programmed death-1, squamous cell carcinoma, treatment, CSCC, cutaneous squamous cell carcinoma, HLP, hypertrophic lichen planus, KC, keratinocytic cancer, LN, lymph node, PD, programmed death

## Introduction

Cutaneous squamous cell carcinoma (CSCC) arising within hypertrophic lichen planus (HLP) is rare,^1^, ^2^, ^3^, ^4^, ^5^ with only 3 cases reported with regional or distant metastasis in the past 20 years (Table I).^1^^,^^2^ Programmed death (PD)1 inhibitors are approved for advanced CSCC, but their usage is not reported in CSCC arising within HLP.

**Table I:** Literature search of cutaneous squamous cell carcinoma (CSCC) arising within hypertrophic lichen planus (HLP) from 2000 to 2022 showed only 3 cases of metastasis of CSCC arising within HLP

# of cases	Regional or distant metastasis identified prior to treatment?	Surgery	Additional treatment(s)	Treatment outcome
8	No	Excision or Mohs surgery^1^, ^2^, ^3^, ^4^	None	Disease-free duration ranged from 5 to 156 months^1^, ^2^, ^3^, ^4^
4	No		Not reported	Outcomes not reported^1^; 1 required amputation due to sepsis^3^
1	No	Wide local excision	Adjuvant radiation	Disease free × 8 months^1^
1	Metastatic to inguinal lymph node^2^	Excision with sentinel lymph node biopsy	Cisplatin + paclitaxel	Progressive disease 1 year after chemotherapy^2^
1	Metastatic to inguinal lymph node^5^	Wide local excision	Radiation, acitretin	Disease free × 3 months^5^
1	Metastatic disease to lung^2^	None	Cisplatin + paclitaxel	Death <4 months after presentation^2^

None were treated with programmed death (PD)-1 inhibitor. Keratoacanthomas and SCC arising within mucosal LP were not included.

Furthermore, new lichenoid dermatitis after PD-1 inhibitors for nonkeratinocytic cancers may occur.^6^ Whether PD-1 inhibitors can exacerbate pre-existing LP in keratinocytic cancer (KC) patients is not known. Hence, the risks and benefits of PD-1 inhibitor use in patients with advanced CSCC within HLP are unclear.

Here, we report a patient with synchronous, bilateral CSCCs within beds of chronic HLP of the pre-tibial skin who was treated with the PD-1 inhibitor, cemiplimab, and developed unilateral inguinal lymph node (LN) metastasis as well as LP exacerbation during treatment.

## Case report

An immune-competent man in his 50s presented with biopsy-proven bilaterally recurrent CSCCs within chronic bilateral pretibial HLP. The biopsy-proven HLP appeared 15 years prior to presentation and was treated intermittently with topical and intralesional steroids.

His first CSCC appeared 9 years after his HLP diagnosis as a 7-cm left pretibial skin mass without evidence of bony involvement on magnetic resonance imaging, and it was completely excised. About 5 years later, the CSCC recurred as a 1-cm left pretibial mass within the surgical scar. Concurrently, a 3-cm right pretibial skin mass was observed, and biopsy confirmed CSCC. Both CSCCs were excised. He continued to treat his HLP flares with topical and intralesional steroids, with serial photography to track his responses.

**Fig 1:**
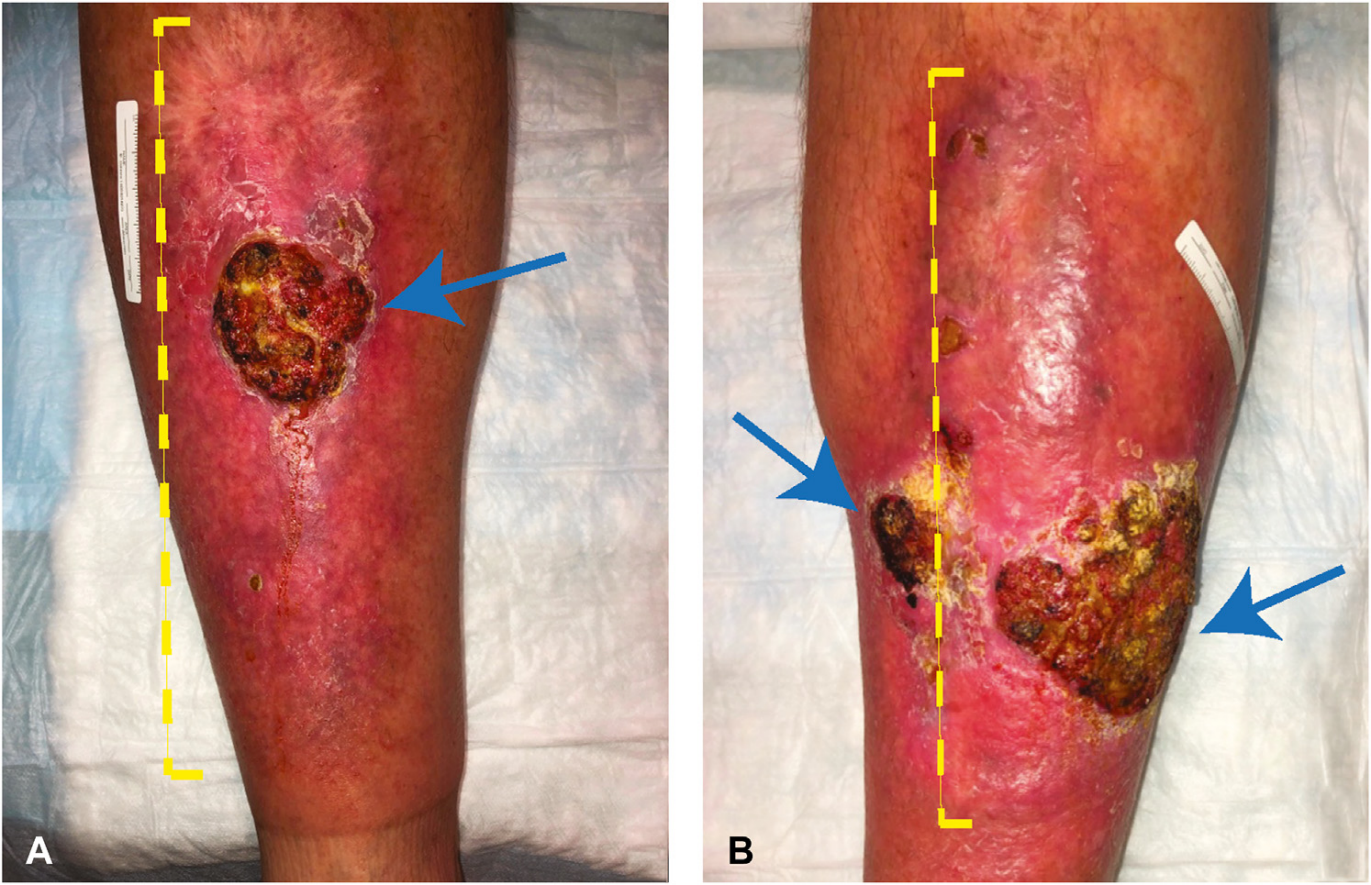
Bilateral, synchronous, biopsy-proven cutaneous squamous cell carcinomas (CSCCs) on the left and right pretibial skin (*arrows*) in a patient with chronic hypertrophic lichen planus (HLP) (*yellow brackets*). **A,** Right and (**B**) left leg.

**Fig 2:**
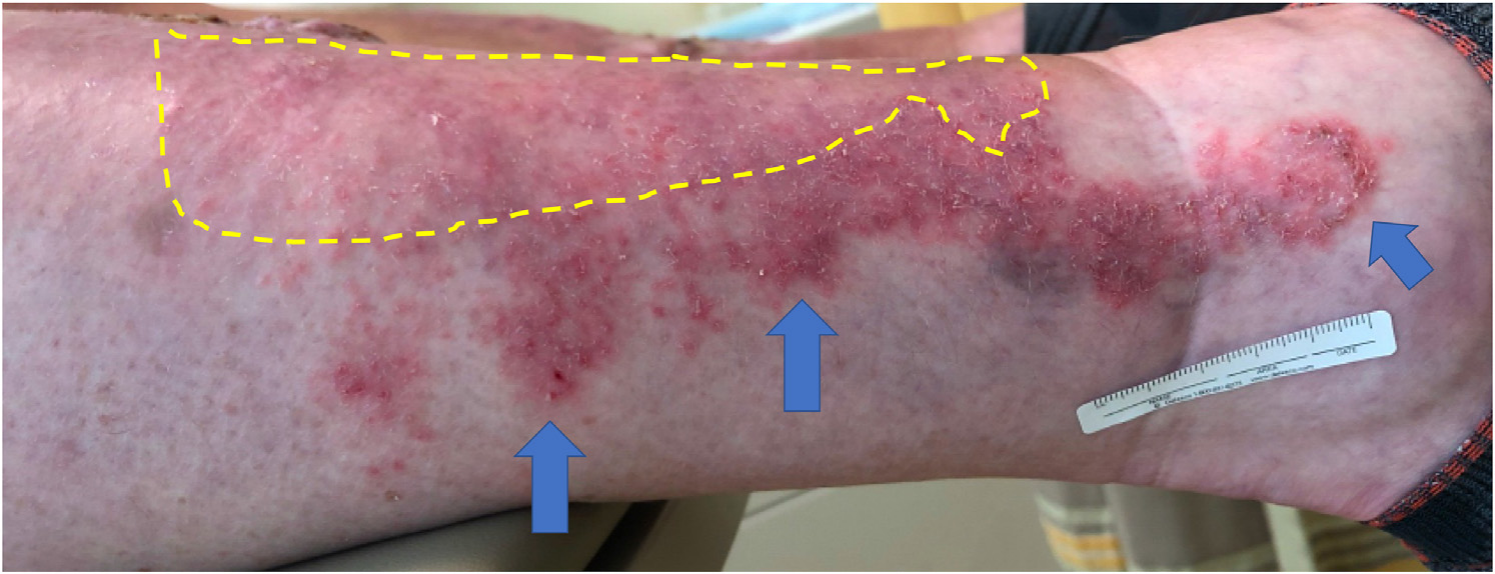
Exacerbation of pre-existing pre-tibial lichen planus (LP) after 4 months of cemiplimab. *Yellow dotted lines* on the right lower leg indicate approximate areas of biopsy-proven LP prior to cemiplimab; *blue arrows* indicate new areas of dermatitis. Similar areas on the left lower leg were biopsied and confirmed lichenoid dermatitis. The patient was prescribed a medium-potency topical steroid to manage the LP.

About 1 year later, the patient presented with new plaques, 2 on the left and 1 on the right pretibial skin, all >2 cm diameter and all <1 cm of his prior CSCCs on both sides. Again, all the CSCCs were within HLP (Fig 1). At this time, right inguinal lymphadenopathy was palpable, and positron emission tomography/computed tomography scans demonstrated an isolated positive signal in that location. A right inguinal LN fine needle aspiration failed to demonstrate malignancy. Multidisciplinary tumor board recommendations were as follows: (1) repeat surgery (which would be bilateral and extensive), or (2) systemic immunotherapy with the PD-1 inhibitor (with risk of serious immune-related adverse events, including cutaneous toxicity). The patient declined surgery and started cemiplimab. While on cemiplimab, the HLP plaques enlarged on both legs (Fig 2), and he restarted a topical steroid.

Unfortunately, the patient’s right inguinal LN continued to grow while on cemiplimab, and repeat biopsy then demonstrated CSCC. However, the right pretibial CSCC distal to the involved LN had nearly completely resolved after 10 months of cemiplimab. The left shin CSCCs remained unresponsive to cemiplimab. He consented to bilateral inguinal lymphadenectomy (which did not show any other involved nodes) but declined surgical excision of the CSCC of the legs. Scout skin biopsies of the right shin were negative for malignancy. A repeat positron emission tomography/computed tomography did not show any areas of concern above the knees.

**Fig 3:**
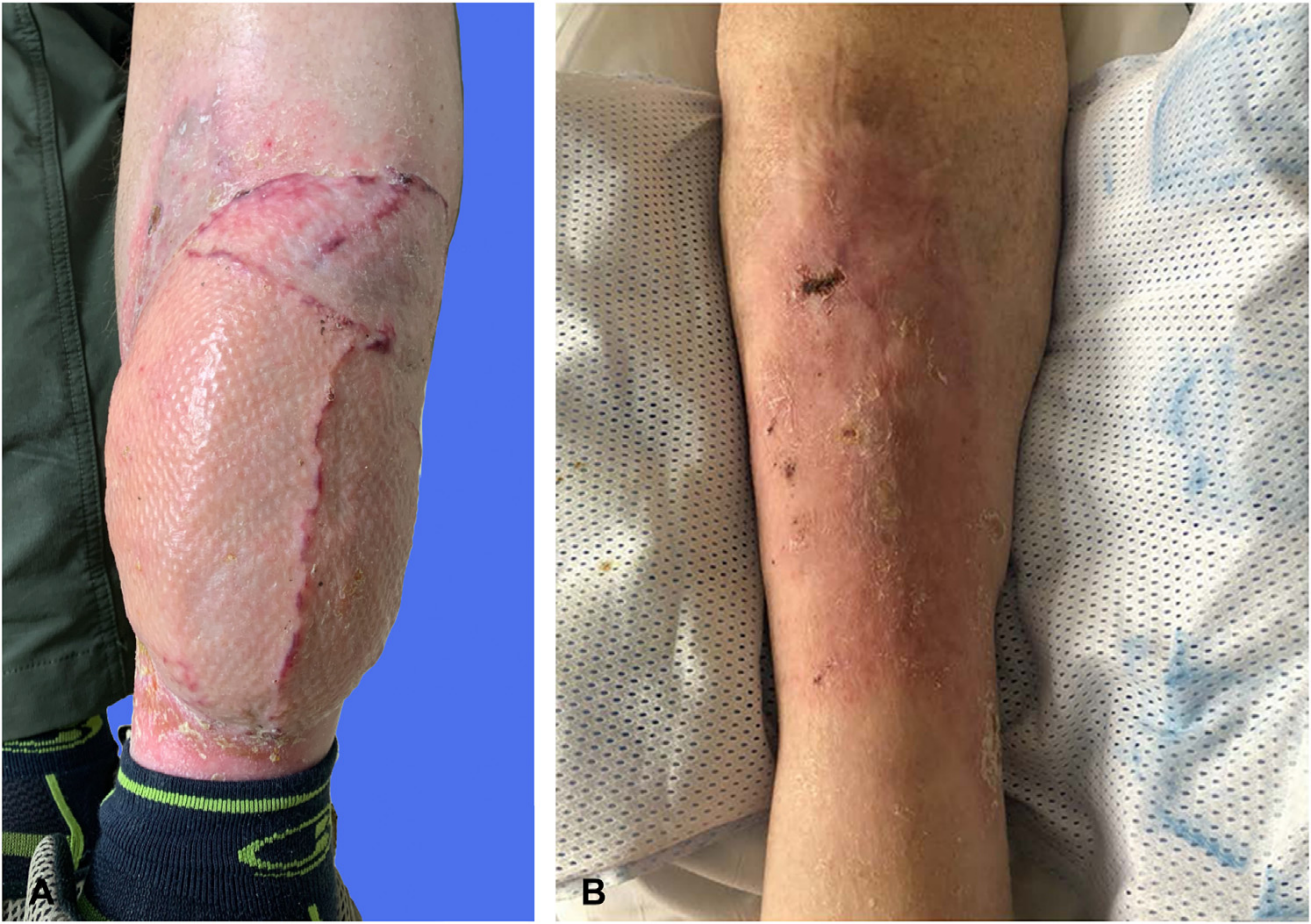
Differential responses of cutaneous squamous cell carcinomas (CSCCs) to cemiplimab in the right versus left pretibial skin. **A,** The left leg CSCCs did not respond to cemiplimab and required surgical resection with soft tissue reconstruction (photograph taken 10 weeks after reconstructive surgery). **B,** The right leg CSCC responded to cemiplimab, with negative scout biopsies, and no surgery was required, although the hypertrophic lichen planus remained.

About 1 month later, the patient experienced acute onset of fatigue with hyponatremia and was diagnosed with severe immune-related adrenal insufficiency, likely due to cemiplimab. He required treatment with systemic steroids and discontinued cemiplimab. He consented to excision of the remaining left pretibial CSCCs, with reconstruction using a free vastus lateralis muscle flap and skin graft placement (Fig 3).

The patient remained disease-free 52 weeks after his last procedure and continues with regular skin and LN surveillance for recurrence. His HLP has returned to pre-cemiplimab severity, and topical tacrolimus will be considered as future therapy if he flares.

## Discussion

This case demonstrated a mixed response to cemiplimab in a patient with multifocal and recurrent CSCC arising within HLP, with nodal metastasis. Despite the cemiplimab-associated adverse events of worsening LP and severe adrenal insufficiency, the patient derived clinical benefit as he was able to avoid extensive surgery on his right lower leg, due to resolution of the CSCC on that side.

This case is instructive on multiple levels. First, metastasis to LNs can occur in CSCC arising within HLP, although the rate of metastasis is not clear due to the lack of systematic study. The extent to which HLP contributed to recurrence and size of the CSCCs is also unclear. Nevertheless, patients may benefit from clinical LN examination, with imaging and/or biopsy if clinically suspicious for involvement.

Second, differential responses to cemiplimab between the primary right pretibial CSCC and the draining right inguinal LN can occur, with the former responsive and the latter not, possibly due to mutational diversity within the source CSCC.

Third, cemiplimab for CSCC may worsen pre-existing lichenoid dermatitis such as HLP but does not necessitate immunotherapy dose hold unless severe. Mild-to-moderate dermatitis can be treated with topical steroids (per American Joint Commission on Cancer [ninth edition] guidelines). While lichenoid dermatitis after PD-1 inhibitors has been associated with favorable oncologic outcomes in a variety of non-KCs,^6^ the implications of *de novo* or worsening of pre-existing LP in KC patients needs systematic study. Also, the effect of topical steroids near the KC on antitumor response of cemiplimab is not well known.

Fourth, clinical trials are underway to use PD-1 inhibitors as a neoadjuvant prior to excision.^7^ While we did not intend to use cemiplimab as a neoadjuvant, this case demonstrates the real-world clinical necessity of multiple treatment modalities (eg, PD-1 inhibitor and excisions) to gain control of aggressive CSCCs.

Finally, CSCC pathogenesis within HLP is not well-understood; however, the bilaterality of the CSCCs within HLP suggests a causal role. More research is needed to confirm if this link is unique to this patient or is generalizable to others. Recent data from LP samples have found altered expression of human endogenous retroviral sequences and apolipoprotein B mRNA-editing enzyme catalytic polypeptide (APOBEC).^8^ High levels of APOBEC-related mutagenesis associate with favorable outcomes after PD-1 inhibition in nonmelanoma tumors.^9^ Translational research is needed to better identify CSCCs likely to respond to immunotherapy, so that other modalities can be used to treat refractory disease.

## Conflicts of interest

ALSC has been a clinical investigator and advisory board member for Regeneron and Merck.
